# Ethical and social insights into synthetic biology: predicting research fronts in the post-COVID-19 era

**DOI:** 10.3389/fbioe.2023.1085797

**Published:** 2023-05-19

**Authors:** Gaofeng Wang, Qingqing Kong, Dong Wang, Fahad Asmi

**Affiliations:** School of Humanities and Social Sciences, University of Science and Technology of China, Hefei, Anhui Province, China

**Keywords:** synthetic biology, social sciences, bioethical concerns, playing God, biosecurity, biosafety

## Abstract

As a revolutionary biological science and technology, synthetic biology has already spread its influence from natural sciences to humanities and social sciences by introducing biosafety, biosecurity, and ethical issues to society. The current study aims to elaborate the intellectual bases and research front of the synthetic biology field in the sphere of philosophy, ethics, and social sciences, with knowledge mapping and bibliometric methods. The literature records from the Social Sciences Citation Index and Arts & Humanities Citation Index in the Web of Science Core Collection from 1982 to 2021 were collected and analyzed to illustrate the intellectual structure of philosophical, ethical, and social research of synthetic biology. This study profiled the hotspots of research focus on its governance, philosophical and ethical concerns, and relevant technologies. This study offers clues and enlightenment for the stakeholders and researchers to follow the progress of this emerging discipline and technology and to understand the cutting-edge ideas and future form of this field, which takes on greater significance in the post-COVID-19 era.

## 1 Introduction

Life sciences experienced three major revolutions since the 20th century (Sharp et al., 2011), the first revolution featuring cellular and molecular biology began with the discovery of the DNA double-helix structure in the 1950s, the second is the genomic revolution, beginning with the exploration of the entire genome of organisms after 2000, and the third is convergence revolution marked by the merging of life science, physical sciences, and engineering (National Research Council, 2014). Synthetic biology (SynBio) serves as one of the most representative convergent disciplines following the idea aforementioned. It has evolved over a huge spectrum of the interdisciplinary continuum which includes life-science branches, such as biochemistry, microbiology, molecular biology, systematic biology, and non-life-science branches like computer sciences and engineering sciences.

Due to its abundant upstream knowledge sources from these disciplines, SynBio has been understood and practiced divergently from the perspective of the respective discipline. For scientists in the bioengineering field, SynBio is defined as “the engineering-driven building of increasingly complex biological entities for novel applications” (Heinemann and Panke, 2006). Luis Serrano (2007) put forward that a group of European experts defined SynBio together as “the engineering of biology: the synthesis of complex, biologically based (or inspired) systems, which display functions that do not exist in nature,” and distinguished SynBio from systems biology by “engineering” and “synthesis of novel functions.” Meanwhile, in the conceptual framework of Gilbert et al. (2010), there are two approaches in SynBio research, the top–bottom approach stressing the function modification of existing cells and the bottom–up approach, which is “interested in the construction of artificial systems” like protocells from a chemical perspective. SynBio in the chemical context is usually emphasized as a science providing microbial chassis and biological devices to explore the interaction mechanism of natural products and yield them which sometimes serves as part of the chemical synthesis, i.e., synthetic chemistry (Keasling, 2008; Goss et al., 2012). Gómez-Tatay and Hernández-Andreu (2019) also stressed the constructive and non-naturally existing feature of SynBio products with the goal that “aims toward the creation of something fundamentally new, biological parts or systems not otherwise found in nature,” which is maintained with a bottom–up strategy.

Above all, most definitions emphasized both the construction of novel biological entities and the modification of existing ones, and a group of leading scientists summarized it as “the design and construction of novel biological parts, devices, and systems, as well as the redesign of existing natural biological systems, for useful purposes” (Calvert, 2010). This definition distinguishes SynBio from other biological disciplines that obtained tremendous scientific gains by exploring the structure and function of naturally existing organisms and entities with a “top–down” strategy locally and deconstructively with a disparate moral status from SynBio, like biochemistry, systematic biology, genetic engineering, and its genetic modification technologies. SynBio emerged as a new concern by its novelty of productions which does not exist naturally or previously, causing uncertainties in ethical issues and biosafety and biosecurity concerns. Compared to the enormous social and ethical discussions on the “top–down” life-science sub-issues like genetically modified organisms (GMOs) (Dong et al., 2019; Nawaz et al., 2019; Zhou et al., 2019), SynBio serves as a novel and probably crucial issue for sociologists, ethicists, and stakeholders. The world has witnessed many breakthroughs in various fields, such as biomedicine and the energy industry with SynBio’s support (Martin et al., 2003; Lindahl et al., 2006). SynBio is used to design DNA-based information storage and communication and processing systems for genetic coding (Cello et al., 2002; Weber and Fussenegger, 2012). In industry, engineered microbial cells were used for biofuel production (Radakovits et al., 2010). In addition to all the advances in the laboratory, additional applications and commercially available products have been made including the chemicals produced by engineered cells and the engineered cells themselves (Voigt, 2020) and more possibilities await. Generally, SynBio appears to be a game-changer for its prospective tremendous potential.

However, despite all of the findings and commercial products SynBio brings to human beings, it also comes with risks and concerns as dual-use research. SynBio’s products are non-natural artificial life with an endogenous uncertainty. Before its emergence, human beings have no experience or knowledge of its existence and nature, and this product itself may have the possibility of self-reproduction and genetic evolution, which could magnify the uncertainty dramatically. This uncertainty itself implies biosafety and biosecurity risks that could cause harm to human beings, organisms, or living environment and leads to psychological and cognitive risks at the individual and social levels, as well as other significant impacts on human society. Therefore, SynBio has also become the research object in humanities and social sciences, attracting wide attention from researchers and stakeholders in philosophy and ethics, policy and laws, science communication and public understanding, and intellectual property. As ethical concerns, SynBio prompts us to think about the intrinsic value of life (Link, 2013), the dignity of life, the integrity of nature, and the relationship between God and His creation (Heavey, 2013). Meanwhile, the novelty of SynBio undermined the feasibility of previous policies or laws for biological or bioengineering practices and appealed for new governance methods and ethical regulations. Some researchers are concerned about SynBio from the public point of view and tested the relationship with more information and deliberation and public support (Kronberger et al., 2012). Moreover, the intellectual property and commercialization of SynBio have also become important research areas and issues for sociologists (Calvert, 2008). All these demonstrate that SynBio has had a significant impact on humanities, featuring philosophy and ethics regarding SynBio and social science research, and would further spread to human society globally.

Meanwhile, in the context of the significant influence of global pandemics such as COVID-19, H1N1, SARS, and MERS on people (Lakoff and Collier, 2008), the public’s concern about the development of novel biotechnology will be magnified significantly. Thus, in the post-COVID-19 era, while SynBio, as a dual-use discipline (Gronvall, 2014), can provide the world with more possibilities and latent enormous benefits, it may encounter new possible risks and obstacles that are concomitant with its emergence and progression as it challenges the long-standing norms and culture, along with the nature, safety, and even existence of human beings. These challenges are mainly revealed, examined, and analyzed by sociologists, ethicists, and philosophers, and are probably overcome as a result of their significant contributions. Therefore, it is necessary to systematically review and summarize the research in humanities, especially philosophical and ethical domains, and social sciences concerning SynBio and elaborate the research fronts (“an emergent and transient grouping of concepts and underlying research issues” (Chen, 2006), which can be conceptualized as the research themes of the collected literature and can be operationally extracted from the keywords and titles of the literature) and intellectual bases (conceptualized as the scientific publications cited by research-front concepts according to Chen (2006)) of this field to provide some references for future research on public understanding, ethics, and governance of SynBio. Some studies have attempted to review the natural science progress of SynBio or argued on partial aspects of its social sciences so far. For instance, Shapira et al.’s (2017) reviewed the SynBio research in the sphere of natural sciences and provided the practical definition and conceptual boundary of SynBio in the study, which significantly helped in collecting the literature. Hayry (2017) elaborated on ethical concerns related to SynBio and its relatedness to GMOs. Similarly, Patrick Heavey (2017) underlined the moral perspective, integrity of nature, and essence of spirituality (Heavey, 2013).

However, there is little research that has hitherto reviewed the entire pool of SynBio social science literature, instead of the natural science ones. In consideration of the huge amounts of research articles for review, traditional reviews are either unable to avoid bias, subjectivity, and incompleteness or time consuming and lack the diversity of analysis methods (Daim et al., 2006). Therefore, in the current research, we introduce bibliometric methods, co-word analysis, co-citation analysis, and some other algorithms to analyze the literature systematically and holistically and use knowledge mapping and visualization technology to provide intuitive and comprehensible insights and conclusions. Specifically, a set of favorable bibliometric software such as CiteSpace and VOSviewer is used for analysis.

Above all, the current study aims to solve the following questions:

RQ1 (Research Question 1): Generally, what does the literature distribution look like? Its aim is to illustrate the yearly growth status, the distribution of the literature at country and institution levels, and the dominant research areas of all the regarding literature.

RQ2 What are the intellectual bases of this area?

RQ3 What are the research hotspots in this research area?

RQ4 What are the potential research trends of SynBio social science studies?

## 2 Methodology

### 2.1 Bibliometrics, co-occurrence analysis, and visualization

In the current research, bibliometric analysis methods were used to illustrate and explicitly describe the evolution path and knowledge structure of SynBio literature in the social, philosophical, and ethical spheres. Bibliometric approaches, referring to the measurement of text and information, help understand the pattern of literature growth, highlight the remarkable research categories, productive research units such as researchers, institutions, and countries, and impactful literature and references. Instead of the partial understanding of certain areas from individual views, bibliometric methods provide a bigger and neutral image of the whole area, specifically the pool of the literature herein, which was later upgraded by Moss’s Database Information Visualization and Analysis (DIVA) system (Morris et al., 2002), which combines bibliometric data with visualization techniques to assist researchers in understanding the scientific literature systematically and visually. Moreover, co-occurrence analysis, including co-word analysis, co-citation analysis, and collaboration analysis, works as a powerful and mature method for detecting the knowledge structure in recent years.

The co-word analysis of keywords is to generate the networks of keywords based on their co-occurrence relationship in the same literature, which implies their similarity and relevance at the level of meaning and helps explore the thematic evolution, research fronts, and trends of a given research field (Cobo et al., 2011). Co-citation analysis has been proven as a well-known approach for intellectual structure detection (Chen, 2006), functions better when the co-citation networks are well clustered (Chen et al., 2010), and can be used to predict the future/emerging trends (Van Eck and Waltman. 2010). Given that SynBio is highly interdisciplinary and dominated by highly specialized research themes, it is much more convenient to find out research gaps among its divergent intellectual knowledge bases with a co-citation approach. In recent years, visualization techniques have been used in co-occurrence analysis, the target units in co-occurrence networks such as cited references, keywords exist as nodes, and the co-occurrence relationship between the units is exhibited as edges that connected the nodes; then, all the target units can be shown together in an informative visual network.

Several tools and software have been developed to analyze and visualize bibliometric data in the last few years, such as CiteSpace and VOSviewer. CiteSpace is a Java software designed for analyzing and visualizing co-occurrence data (Chen, 2017) that is especially good at co-citation analysis, which facilitates the analysis of emerging trends and transient patterns in the scientific literature (Chen, 2006). VOSviewer was developed for creating, visualizing, and exploring bibliometric maps of science and good at text mining and visualization (Van Eck and Waltman. 2011). This study uses VOSviewer and CiteSpace, two mature visualization tools with respective advantages, to analyze the SynBio literature in the sphere of social sciences.

### 2.2 Data collection

This study focuses on the original and impactful research of SynBio in social, philosophical, and ethical domains, so we collected the article and reviewed records from the Web of Science Core Collection, including SSCI and AHCI, as a data source. To eliminate the risk of skewness from collecting records, the conference proceedings, letters, retracted manuscripts, and book chapters were excluded.

Owing to its rich connotation and without a well-accepted definition of SynBio, the relevant terms for retrieving need to be organized systematically. To confront the challenges of dealing with interdisciplinary definitions (Kuzhabekova and Kuzma, 2014), many scholars adopted expert-defined keyword methods, such as Porter et al. (2008); Small et al. (2014), or other semi-automatic retrieving methods. To cope with the validity- and reliability-related challenges, we cited the concept system of SynBio from the work of Shapira et al.’s (2017) and upgraded and created a novel definition system of SynBio according to the relevant elements or definitions of SynBio in the social science context. For instance, we removed the terms which refer to cell phones instead of artificial cells for disambiguation and added more terms about SynBio like “protocell” and “DIY biology.” DIY biology refers to do-it-yourself biology (DIYbio), and several DIYbio groups have formed from the encounter of amateur sciences with synthetic biology (Seyfried et al., 2014). As involved with the high level of publicity and engagement of public by its very definition, DIYbio has been catching the attention of ethical and social researchers from the outset with plenty of social science articles published and has been held accountable for the rise of biohacking (Meyer and Vergnaud, 2020). Moreover, since we need to examine the research regarding SynBio rather than the long-standing debate on GMO, which is distinguished from SynBio by the definition mentioned above, the concepts closely related to GMO were excluded like “transgen*,” “genetic engineering,” and “genetically modified,” so as to the general technology used in GMO and SynBio like “gene editing” and “CRISPR.” Eventually, 428 article records and 2,474 references were collected on 24 April 2021. The process of creating the definition system of SynBio is shown in Table 1.

**TABLE 1:** Search strategy.

Step	Operation	Result
1	Search in the SSCI and AHCI based on the search strategy offered by Shapira et al.’s (2017) and check the search results	Found many irrelevant records due to improper searching terms such as “artificial * cell phone”
2	Search the terms on SynBio provided by Shapira one by one in the SSCI and AHCI and check the results; remove the terms with irrelevant records and maintain the effective ones	“Synthetic biolog *” “synthetic DNA” “synthetic genom*” “synthetic gene”
3	Add relevant terms from the retrieved literature and other experts’ definitions of SynBio and test the terms in turn	New keywords: “synthetic cell” “artificial cell” “do-it-yourself biology *” “DIYbio” “DIY biology *” “biobrick” “protocell virus” and “protocell"
4	The formula for the search strategy with the terms mentioned above and Boolean operators, and remove the irrelevant ones with the Boolean operator “NOT”	The final search strategy is as follows: TS=(“synthetic biology*” or “synthetic DNA” or “synthetic genom*” or “synthetic gene” or “synthetic cell” or “artificial cell” or “do-it-yourself biology*” or “DIYbio” or “DIY biology*” or “biobrick”) or (TS= (“artificial life” or “artificial lives”) and TS=(protocell or virus) or [TS=(protocell) NOT TS=(“dichroic reflector” or architecture)]
	Combine the keywords obtained by the two methods and conduct the fourth screening to obtain the final search formula	

## 3 Empirical results

### 3.1 RQ1: literature distribution

**FIGURE 1:**
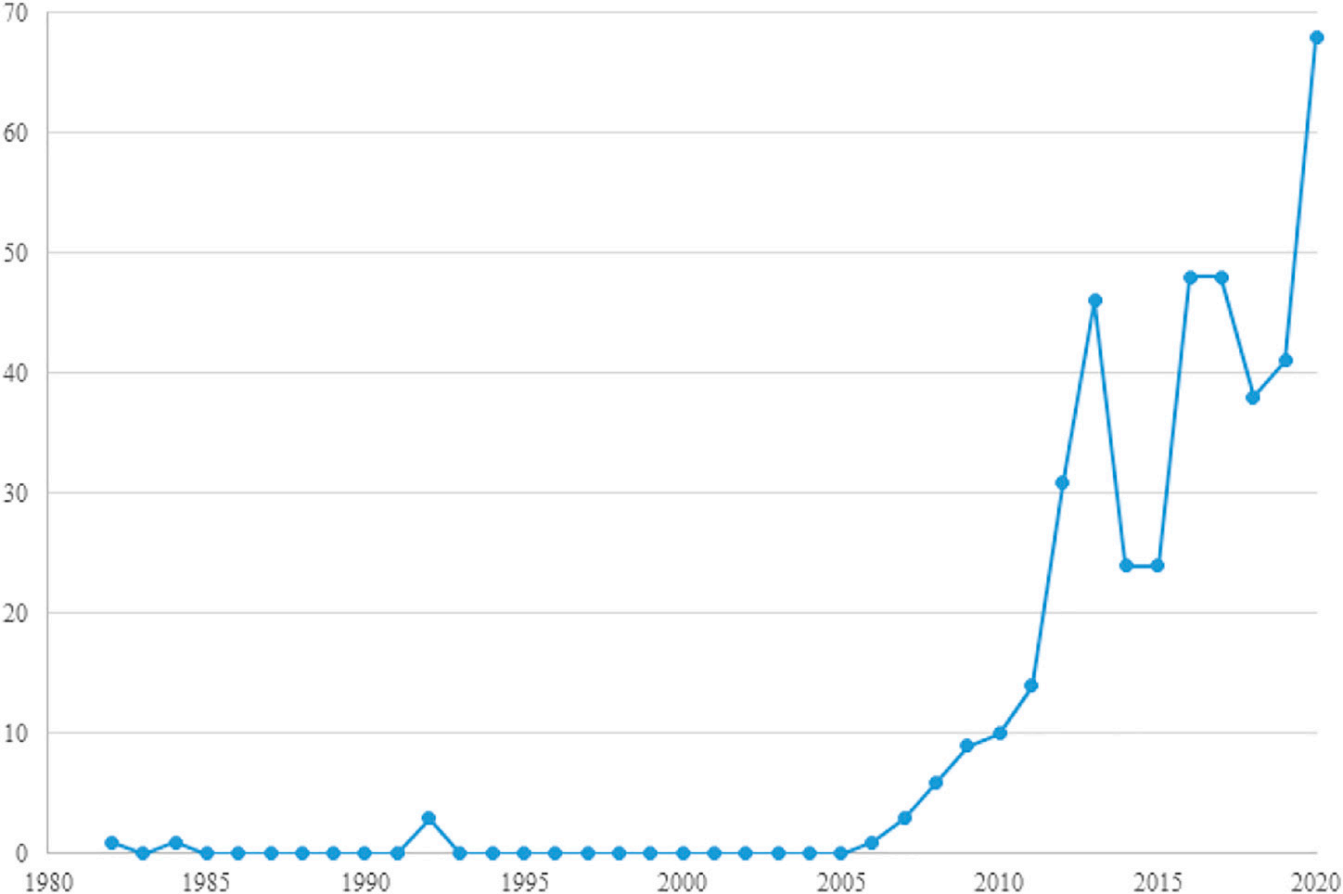
Yearly publication records of the SynBio literature in social sciences.

The number of the literature grew in succession from 2006 to 2020 despite the slight vibration, and the peak appears in 2020, which implies the increasing and consistent attention from social and ethical researchers, so there may be more research oncoming. As the data of total publications of 2021 was not available when it was collected and the incomplete data might impede the understanding of the publishing trend, therefore the data for 2021 is excluded here. Figure 1 shows the yearly growth of publication records. The earliest record in this collection is “Social Responsibility in an Age of Synthetic Biology” by Sheldon Krimsky (1982), which highlighted the risk of rDNA as a bioweapon and appealed to harness rDNA technology and the social responsibility of SynBio research to society earlier then the emergence of the first artificial cell “Synthia” (Gibson et al., 2010), which represented the early concerns on SynBio. Since 2006, consecutive articles are published owing to the bioweapon concerns like synthetic virus genome and other ethical issues on SynBio, and it manifests the continuous attention on SynBio from social and philosophical spheres. The first surge occurred in 2010–2013, right after the growth of the first synthetic cell (Gibson et al., 2010) which made a huge stir globally. It has such a profound impact that we believe this event directly led to this sharp spike. Hereafter, the publication number went up and down as the novel development of SynBio emerged worldwide like the invention of CRISPR technology in 2013 (Cong et al., 2013), the first synthetic minimal cell (Hutchison et al., 2016), the first synthetic yeast genome (Richardson et al., 2017), and so on.

#### 3.1.1 Most productive countries and institutions

The list of the top productive countries is dominated by developed countries (Table 2). Specifically, two English-speaking countries, i.e., the US and UK, took the top two positions and account for over 50% of the total publications, which are followed by Germany, the Netherlands, France, and other European countries. While this result is in line with the advances in the lab of SynBio research and development happening in the developed countries like the United States and European countries, the enormous endeavor like the reflection of social and philosophical impact of SynBio and deliberations, conferences, and policies regarding this novel biotechnology account for the prosperity and productivity in publications of these countries in a way. Some of the policies have been put into effect such as *New Directions: The Ethics of Synthetic Biology and Emerging Technologies* released in 2010 by the Presidential Commission for the Study of Bioethical Issues in the United States and *Final Opinion on Synthetic Biology III: Risks to the environment and biodiversity related to synthetic biology and research priorities in the field of synthetic biology* released in 2015 by European Commission—Scientific Committees. Additionally, it should be noted that the current research mainly follows the opinions published in articles by social and philosophical researchers rather than public opinion in massive or social media which can be probed better in the perspective of communications. Furthermore, we chose only the SSCI and AHCI as data sources, which may not draw on some articles outside the index, especially the ones from non-English-speaking countries. Therefore, the developed countries might become overrepresentative when looking into the SynBio social research.

**TABLE 2:** Publication records of top prolific countries or regions.

Rank	Country/region	Publication	Proportion (%)
1	United States	126	29.439
2	England	83	19.393
3	Germany	36	8.411
4	The Netherlands	33	7.71
5	France	30	7.009
6	Spain	30	7.009
7	Scotland	27	6.308
8	Switzerland	24	5.607
9	Austria	20	4.673
10	Canada	18	4.206

**FIGURE 2:**
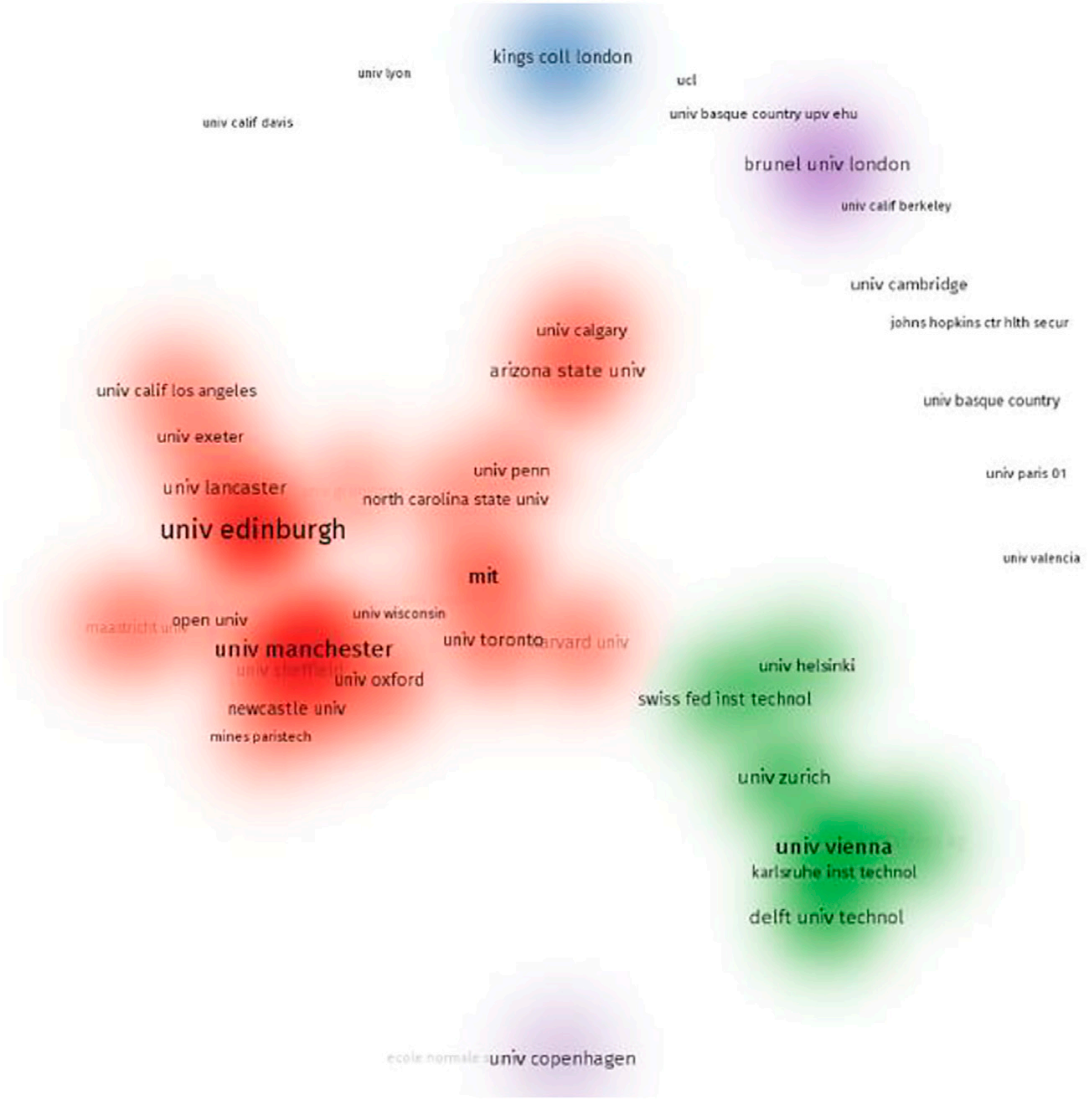
Institution collaboration networks in this field.

**FIGURE 3:**
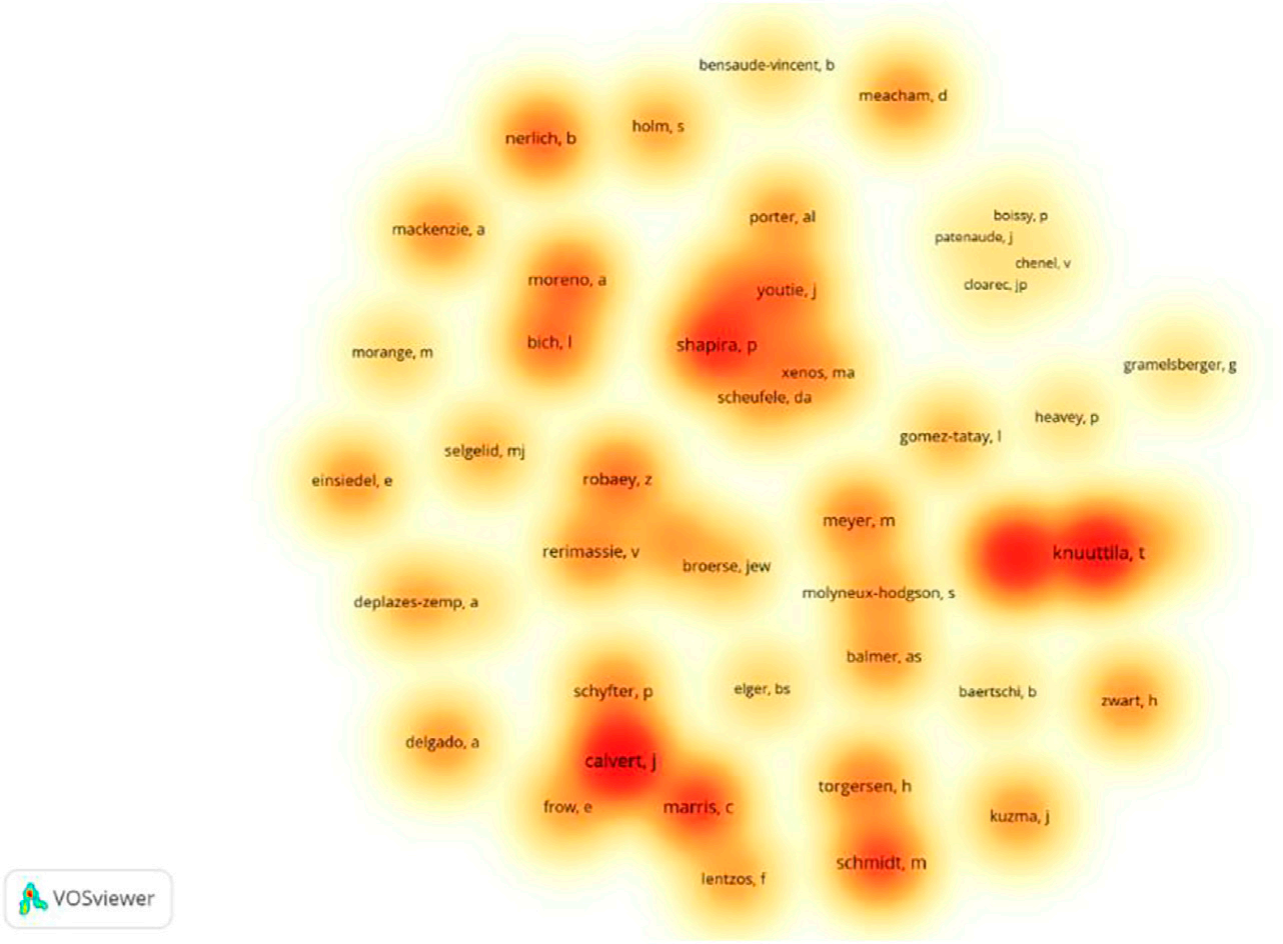
Author collaboration networks in this field.

The most prolific institutes include the University of Edinburgh (25 records), the University of Manchester (18), the Georgia Institute of Technology (10), the University of Copenhagen (9), and the University of Zurich (9). British universities are distinguished from all the contributors. Professor Shapira of the University of Manchester is also affiliated with the Georgia Institute of Technology, and these two institutions share the publication records. To understand the collaboration among institutions, VOSviewer was used to illustrate their co-occurrence networks here (Figure 2). We use the density visualization function in VOSviewer to mark the most productive institutions and their collaborations, in which two major clusters are generated in red and green color. Two research teams in the UK are closely internally connected in their network, led by the University of Edinburgh, with the University of Lancaster and the University of Exeter, and led by the University of Manchester, with the University of Sheffield and the University of Oxford. Moreover, in the red cluster, several universities from the United States are also well connected, including the Massachusetts Institute of Technology, the University of California at Los Angeles, the University of Pennsylvania, and Harvard University. Institutions from continental Europe have also formed networks in the green cluster, including the University of Helsinki, the University of Vienna, the University of Zurich, and some other institutions. With the same approach, we illustrated the collaborative network of researchers in this field (Figure 3), three teams standout, including Calvert’s team at the University of Edinburgh, Shapira’s team at the University of Manchester, and Knuuttila’s team at the University of Vienna.

#### 3.1.2 Research areas

In this field, the research area with the most publications is the history and philosophy of science (Table 3), followed by ethics, social sciences biomedical, and philosophy. Evidently, amid the top 10 research areas, the philosophical and ethical associated issues (including #1, #2, #4, and #7 areas) serve as the dominant theme of this field and account for 68.46% of the publication records. In addition to the long-standing debates in ethics, philosophy, and social science domains, other areas like environmental studies and communication emerge as novel perspectives to explore the social impact of SynBio.

**TABLE 3:** Top 10 research areas of the SynBio-related social science literature.

Rank	Research area	Number of publications	Proportion (%)
1	History and Philosophy of Science	124	28.972	
2	Ethics	91	21.262	
3	Social Sciences Biomedical	55	12.85	
4	Philosophy	51	11.916	
5	Social Issues	43	10.047	
6	Multidisciplinary Sciences	34	7.944	
7	Environmental Studies	29	6.776	
8	Medical Ethics	27	6.308	
9	Engineering Multidisciplinary	24	5.607	
10	Communication	22	5.14	

### 3.2 RQ2: intellectual bases of the current field

Research fronts and intellectual bases are fundamental concepts in information science, the former focus on the grouping of concepts and underlying research issues, while the latter refers to the citation and co-citation footprint in the scientific literature (Chen, 2006), which helps detect the evolvement and knowledge origin of certain research fields. In this section, we used CiteSpace software to analyze the references of the collected records, especially the co-citation relationship of these references and their source titles.

We found the top highly co-cited references, which are visualized as nodes by the software in the co-cited reference network, and the co-citation relationship of each pair of references is visualized as the edge between the nodes. Then, we conducted a cluster analysis of the co-cited references and labeled the clusters with the embedded algorithm log-likelihood ratio to elaborate the themes of the clustered references within the network. The highly co-cited journals are listed at the end of this section to facilitate the analysis of the intellectual bases. CiteSpace software has several advantages when dealing with co-citation data. First, it can detect highly co-cited references (Table 4) and the nodes with high co-citation frequency in the network. Meanwhile, the nodes in the network can also be clustered according to their spatial distance, so the nodes with details can be classified and labeled as clusters to extract knowledge from detailed information by CiteSpace’s built-in text mining algorithms, such as the log-likelihood ratio whose effectiveness was introduced by Chen et al. (2010).

**TABLE 4:** Top 10 highly co-cited references.

No.	Frequency	Title	Author	Year	DOI
1	46	Creation of a bacterial cell controlled by a chemically synthesized genome	Daniel G. Gibson et al.	2010	10.1126/science.1190719
2	32	Knowledge-making distinctions in synthetic biology	Maureen A. O'Malley et al.	2008	10.1002/bies.20664
3	26	Foundations for engineering biology	Drew Endy	2005	10.1038/nature04342
4	25	Developing a framework for responsible innovation	Jack Stilgoe, et al., 2020	2013	10.1016/j.respol. 2013.05.008
5	19	Synthetic biology and the ethics of knowledge	Thomas Douglas and Julian Savulescu	2010	10.1136/jme. 2010.038232
6	18	That was the synthetic biology that was	Luis Campos	2009	10.1007/978-90-481-2,678-1_2
7	17	Tales of emergence: Synthetic biology as a scientific community in the making	Susan Molyneux-Hodgson and Morgan Meyer	2009	10.1017/S1745855209990019
8	16	Five hard truths for synthetic biology	Roberta Kwok	2010	10.1038/463288a
9	16	Responsible research and innovation: From science in society to science for society, with society	Richard Owen et al.	2012	10.1093/scipol/scs093
10	15	Diffusion of synthetic biology: A challenge to biosafety	Markus Schmidt et al.	2008	10.1007/s11693-008-9018-z

#### 3.2.1 Highly co-cited references related to SynBio in social sciences

We listed the top 10 references (in Table 4) with a high frequency of co-citation, which means they were highly co-cited by the citing articles we collected from the Web of Science, which indicated their high impact as the intellectual base for this field. Only two among the top 10 co-cited references came from the natural science fields, including a review article about the brief history and research progress of SynBio (#3) written by Endy (2005). The other references from the natural science realm with the highest frequency (#1) is the first research of self-replicating bacterial cells using synthetic DNA in 2010, achieved by a team with Venter’s leading (Gibson et al., 2010). This research was a blast appealing to global attention and triggered great concerns throughout natural and non-natural science fields and the public ever since.

Beyond the natural science perspectives, the #2 highly co-cited reference (A. O'Malley et al., 2008) helped explicate the rationale and relationship between synthesis and analysis, which was followed by the discussion of bottom-up and top-down strategies in SynBio research and innovation (Gilbert et al., 2010); distinct SynBio from disciplines like biological engineering and systems biology, meanwhile contributed to locating and delimiting research objects and laying a foundation for further SynBio social and humanities research in respect to the fact that it was published relatively early, and calling for attention on knowledge itself. Subsequently, the # 5 reference (Douglas and Savulescu, 2010) brought about the rethinking of ethics of knowledge in SynBio. It introduced ethics of knowledge into the research on SynBio based on the “misuse of knowledge,” initiated a new research domain, and provoked widespread debate. For instance, Pierce (2012) “challenges(d) an ethics of knowledge to respond to concerns of procedural and substantive justice,” and invited more consideration on the decision by whom, whose interest and effectiveness of ethics of knowledge regarding SynBio.

As early as in 2008 in the #10 reference, Schmidt (2008) highlighted the unprecedented biosafety challenges caused by engineered de-skilling SynBio practices (like garage biology or do-it-yourself biology), especially by newcomers without formal biosafety training including researchers from other disciplines and public, as it may lead to biohackery, illicit bioeconomy, and appealed for appropriate safety standards. In 2009, the #6 reference, a chapter written by Campos (2009), examined the coinage of “synthetic biology” back to 1912 by Stéphane Leduc in his *La Biologie Synthétique* and elucidated the nature and features of SynBio as technology and engineering from a historical perspective. In addition, Molyneux-Hodgson and Meyer (2009) viewed SynBio from a perspective of the scientific community, as the community of SynBio contains not only researchers but also interested citizens distinctively, probed the emergence and four types of formation of the SynBio discipline community, and constructed a framework of “movements” and “stickiness” to understand this community.

Regarding the prevailing framework for the governance, administration, and normalization of SynBio, Owen, Macnaghten, and Stilgoe (in #9 reference) provided a historical overview of the concept and three features of responsible innovation (RI) after the *Horizon 2020 Strategy* was put forward by the EU (Owen et al., 2012). Furthermore, they (in #4 reference) systematically constructed a framework to integrate SynBio and RI, illuminated four integrated dimensions of RI in SynBio research, including anticipation, reflexivity, inclusion, and responsiveness, and discussed the universality of RI in SynBio beyond the use in UK Research Councils and the scientific communities (Stilgoe and Macnaghten, 2013).

For #8, Kwok (2010) introduced five challenges that SynBio had to confront back in 2010 in a Nature news feature and poured cold water on the hypes regarding SynBio’s overrated prospects. This news feature was later criticized by Voigt (2020) as an “infamous article” based on the evidence of commercially available products from SynBio research. The top 10 highly co-cited references not only highlighted diverse dimensions regarding the research and innovation of SynBio but also harnessed most of the substantial issues, especially on the social, philosophical, and ethical fronts.

#### 3.2.2 Intellectual bases: cluster analysis of co-cited references

**FIGURE 4:**
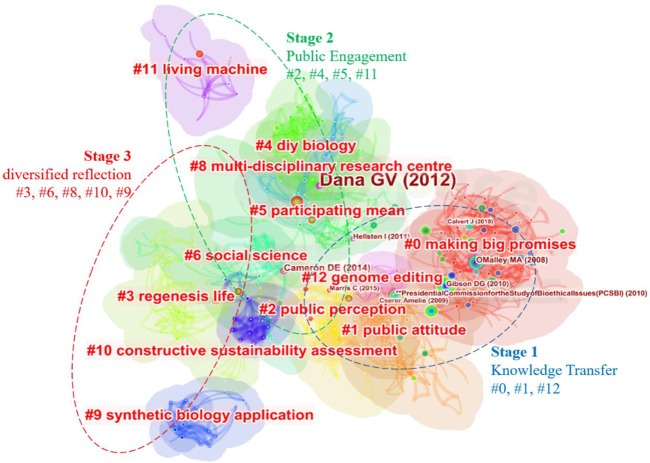
Clusters in the co-cited reference network. Knowledge transfer stage is the earliest one and contains three clusters, including the biggest two clusters (#0 and #1), well connected and located on the right part. In this stage, the concepts and distinctions from relative disciplines (O’Malley et al., 2007), relative technologies, and potential applications of SynBio were widely introduced into humanities and social sciences, as well as philosophical studies, during which SynBio served as a novel issue, and people crave for its bright prospect, commercial value, intellectual property (Calvert, 2008; Saukshmya and Chugh, 2010), and technology potentials. The huge commercial value of SynBio intellectual properties and patents are not completely consistent with the open-source philosophy of SynBio like what happens in International Genetically Engineered Machine (iGEM) competitions, sparking debates on legal issues (Contreras et al., 2015). In addition, some studies focus on the ethical issues of SynBio as well.

All the co-cited references in the network are clustered, labeled, and visualized with CiteSpace, and the distribution of each cluster is shown in Figure 4. The clusters are identified by their size (Cluster #0 has the most nodes) and ranked by their mean year (Table 5). The silhouette value indexed the similarity of nodes in the same cluster, and each of them is over 0.7, which indicates the homogeneity of the nodes within the same cluster for analysis. The clusters can be divided into three stages generally based on the mean year of references in each cluster and extracted labels, i.e., the “knowledge transfer” stage (Cluster #0, #1, and #12) during which the concepts, ethical concerns, and knowledge produced in the natural science research flooded into the social and humanities sphere, triggered the knowledge transfer from natural science to social and ethical domains; the “public engagement” stage (Cluster #2, #4, #5, and #11) featuring public engagement such as DIYbio and public participation as major topics; and the “diversified reflection” stage (Cluster #3, #6, #8, #10, and #9) which contains not only the application and impact of SynBio but also the assessment and reflection on SynBio itself.

**TABLE 5:** Size, silhouette, and labels of each cluster in the co-cited reference network.

Stage	Cluster-ID	Size	Silhouette	Mean (year)	Label (LLR)
1 knowledge transfer	0	97	0.852	2007	Making big promises
	1	57	0.746	2011	Public attitude
	12	10	0.959	2012	Genome editing
2 public engagement	2	43	0.831	2013	Public perception
	4	41	0.949	2013	DIY biology
	5	35	0.725	2013	Participating mean
	11	13	0.973	2013	Living machine
3 diversified reflection	3	43	0.916	2014	Regenesis life
	6	34	0.944	2014	Social science
	8	24	0.951	2015	Multidisciplinary research center
	10	16	0.934	2015	Constructive sustainability assessment
	9	18	0.962	2016	Synthetic biology application

In the public engagement stage, public perception and public participation became the major concern in this field. No matter the experiments and practices of the public on biological materials with SynBio technology, like DIYbio, or the perception, governance, monitoring, and policymaking on SynBio, public engagement appears as the non-negligible and inevitable issue since the emergence of SynBio itself. The clusters about public participation connected closely in this stage, while the Cluster #11 living machine, located separately from them in the same period, indicates the concerns on the ethical and philosophical discussion about SynBio.

The latest stage “diversified reflection” turned out to be multiple regarding themes, and the researchers reevaluated and reflected on SynBio from divergent perspectives. In addition to the previous issues in the last two stages, novel technologies used in SynBio, innovative administration and management, sustainability assessment, and other various themes supplied various knowledge bases for this field in the latest period. Novel and continuously improving technologies like CRISPR (Heidari et al., 2017) and gene editing are hot topics of controversy in recent years, especially after the “gene-edited babies” event that two baby girls were born, whose genomes were edited using CRISPR/Cas9, a gene-editing tech, during the embryo stage to confer the babies innate resistance to HIV as one of their parent was HIV-positive. The genome-editing research on human embryos aiming to “help people with HIV-related fertility problems” turned out to be associated with unpredictable risks (Wang and Yang, 2019), provoked a fierce global backlash (Cyranoski et al., 2018), and prompted people’s attention to biotechnological ethics. Meanwhile, the social scientists also have conducted research on genome editing for decades (Thompson, 2012) and published piles of articles which provide plenty of important theories and experiences for the ethical reflection on SynBio for reference so that “genome editing” becomes the representative label of references in Cluster #12. These studies reconsidered the general problems that emerged in the development of new technologies, such as the lack of transparent regulatory standards and outdated management regulations, and provide references to avoid similar problems which harass SynBio. The multi-disciplinary research center (Vermeulen, 2018) and its innovative management have also caught much attention in recent years. Especially, “responsible innovation” stands out as one of the promising solutions for the bioethical issues (Vermeulen et al., 2017) of emerging biosciences and biotechnologies, which impacts the daily practices and behaviors of scientists and outcomes of their studies (Pansera et al., 2020).

#### 3.2.3 Highly co-cited journals

Using the same research method as in Section 3.2.2, we obtained the top 20 highly co-cited journal’s table (Table 6), including the journal titles, co-cited frequency of journals, centrality in the network, the mean year of being cited, and the half-life of each journal. It indicated that the early source titles are mainly from the field of natural sciences, while in recent years, the journals from the field of social sciences take the dominant position, and there is a shift in the sources of knowledge in this field. Among all the high-centrality nodes which have structural importance, *Biosocieties*, *BioEssays*, *Social Studies of Science*, *Public Understanding of Science,* and *Research Policy* are the prominent knowledge sources for the development of this field in the social science sphere.

**TABLE 6:** Top 20 highly co-cited journals of the SynBio-related social science literature.

Frequency	Centrality	Cited journal	Mean year	Half-life
258	0.1	Nature	2007	8.5
229	0.05	Science	2007	8.5
149	0.06	Nature Biotechnology	2007	8.5
120	0.07	EMBO Reports	2007	8.5
101	0.1	Proceedings of the National Academy of Sciences	2009	7.5
80	0.24	Biosocieties	2009	5.5
73	0.07	Nature Reviews Genetics	2009	6.5
70	0.08	Molecular Systems Biology	2010	4.5
66	0.11	BioEssays	2009	5.5
66	0.1	Social Studies of Science	2012	4.5
64	0.08	PLOS One	2012	5.5
63	0.02	Science, Technology, & Human Value	2009	7.5
62	0.13	Public Understanding of Science	2012	4.5
59	0.09	Nanoethics	2011	5.5
59	0.03	Science and Engineering Ethics	2012	4.5
57	0.12	Research Policy	2014	3.5
52	0.11	PLOS Biology	2012	3.5
50	0.06	Trends in Biotechnology	2012	6.5
49	0.07	Studies in History and Philosophy of Science Part C: Studies in History and Philosophy of Biological and Biomedical Sciences	2012	4.5
43	0.09	Science and Public Policy	2014	3.5

### 3.3 RQ3: hotspots in the SynBio-related literature in social sciences

**FIGURE 5:**
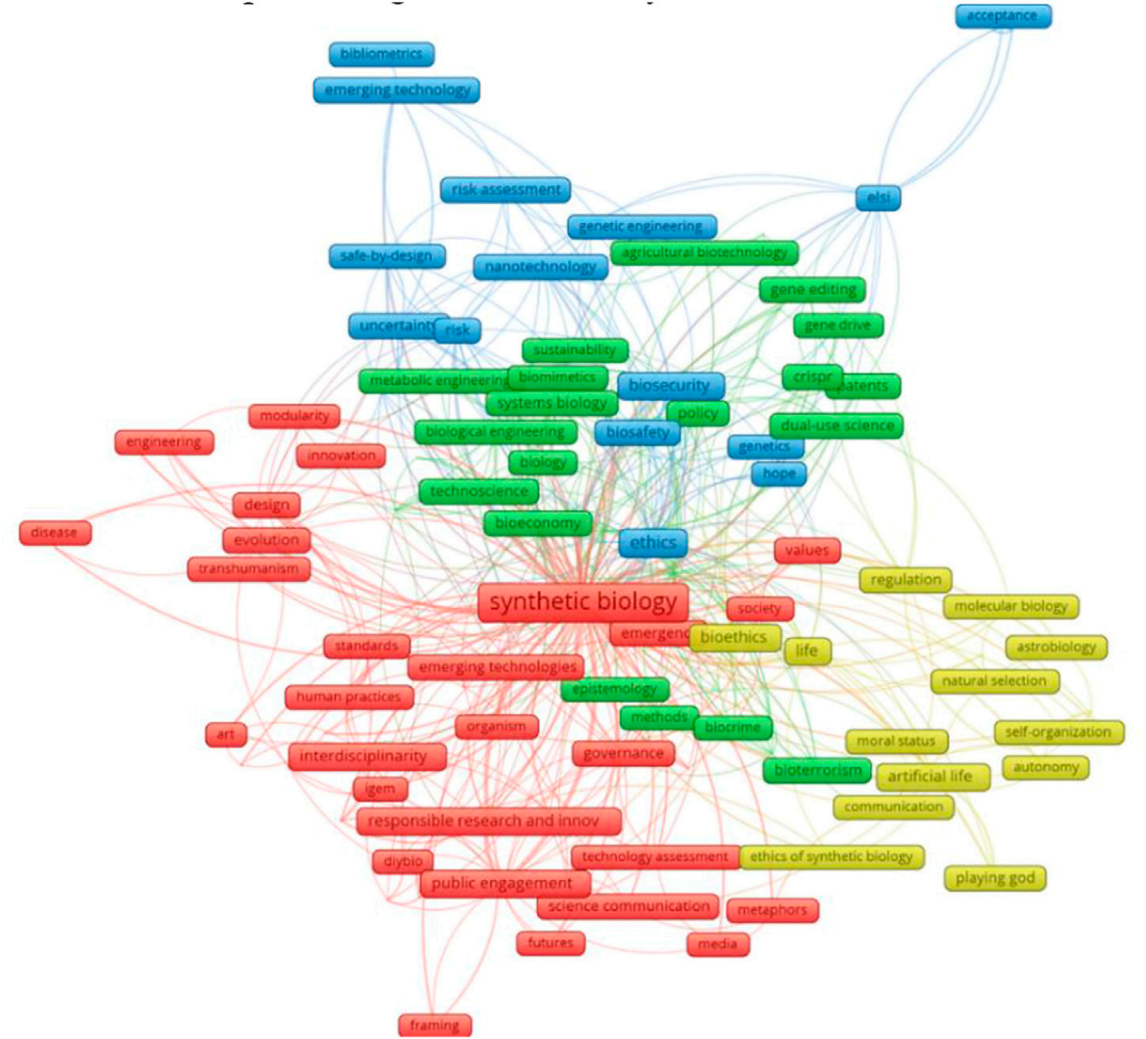
Co-word analysis of the keyword network. “ELSI” refers to Ethical, Legal, and Social Implication; “CRISPR” refers to clustered regularly interspaced short palindromic repeats; “iGEM” refers to International Genetically Engineered Machine; and “DIYbio” refers to do-it-yourself biology.

**FIGURE 6:**
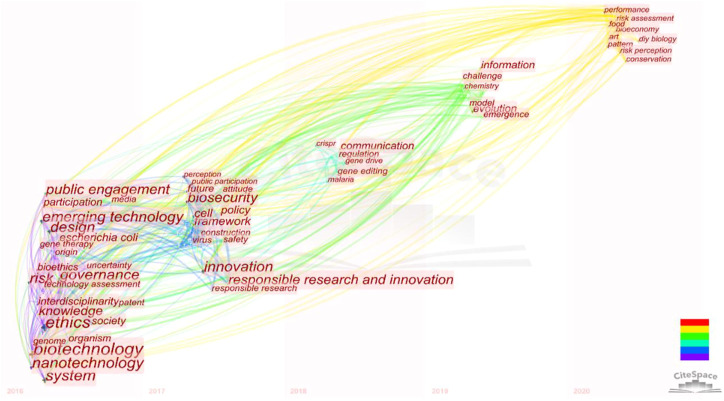
Keyword-based trend analysis to highlight emerging trends and possible future.

In this section, VOSviewer software was utilized to conduct a co-word analysis on the keywords of all the literature collected in this field and visualize the network of co-word relationships, as shown in Figure 5, with the built-in algorithms. In this science mapping practice, keywords in the collected literature are linked according to their co-occurrence relationship, and the keywords with a larger node size (and proportionally larger fonts) have a higher degree which refers to the number of edges and indicates its impact. With the built-in clustering algorithm of the software, four clusters emerged and were marked as red, green, blue, and yellow, as shown in Figure 6, and each cluster represents a sub-theme of the current field.

*Cluster 1 (red):* This cluster represents the themes mainly about the governance of SynBio, containing three aspects of keywords such as assessments and standards, Responsible Research and Innovation (RRI), and public engagement. In addition to the searched terms like “synthetic biology,” other high-impact keywords in this cluster are governance, standards, values, and technology assessment; Responsible Research and Innovation; and public engagement, science communication, media, DIYbio, and iGEM. Technology assessment (TA), a set of wildly used methods for assessment and governance (Trump et al., 2019) of emerging technologies during the 1970s–1990s, aimed to provide an early warning by evaluating emerging technologies so that potential and unexpected negative impacts can be corrected and remedied promptly, while they were more concerned on risk evaluation rather than ethical issues. After that, the Ethics, Legal, and Social Issues (ELSI) program was put forward alongside the Human Genome Project by the National Institute of Health and focused more on the interdisciplinary research and the interaction between different stakeholders in society and refers to the ethics and legitimation of the research studies (Gregorowius and Deplazes-Zemp, 2016). However, the ELSI practice enacted the epistemological gap as a division of labor “where scientists perform science and leave social, moral, and ethical questions to experts–ethicists, theologians, lawyers, and social scientists” and are often based on a simplified linear model of innovation pathways and outcomes (Marris et al., 2015). Then, RI (or RRI) was promoted and supplied an interdisciplinary frame for the governance of SynBio and emphasized public participation in the whole process of innovation, including the assessment of technology itself, offering the idea of social needs and wishes, and decision on the final plan to meet them. Gregorowius and Deplazes-Zemp (2016) pointed out that TA and ELSI research can be regarded as the early development stage and predecessor of RRI. So, in this cluster, all three aspects of keywords refer to the responsible governance of SynBio and can be covered in the frame of RRI.

*Cluster 2 (yellow):* Keywords in this cluster represent the studies mainly related to philosophical issues of synthetic biology, including “moral status,” “playing God,” “artificial life,” “self-organization,” “natural selection,” and bioethics. These studies highlighted the philosophical issues of SynBio and its product, i.e., artificial life. Some religious strong believers oppose SynBio because the creation of artificial organisms by human beings encroaches on a domain of activity that has been considered to be God’s divine prerogative (Dragojlovic and Einsiedel, 2013). Moreover, how to position the moral status of artificial life, and human beings, as peer life and creator, may confront the challenges of moral statuses from artificial life. Moreover, these creation practices substitute for natural selection in the evolution process and lead to new philosophical concerns.

*Cluster 3 (blue):* Keywords in this cluster primarily related to the ethical concerns of SynBio, especially the potential risk and danger that SynBio may bring about. The high-frequency keywords in this cluster are “ethics,” “risk assessment,” “uncertainty,” “ELSI,” “biosafety,” and “biosecurity”. The uncertainty of the process of research and practice on SynBio and its products and the risks they may bring about are related to biosafety and biosecurity at the material level. Gómez-Tatay and Hernández-Andreu (2019) tried to distinguish biosafety from biosecurity, and “biosafety refers to the prevention of the risks to public health and the environment that could be produced by accidental interactions between dangerous biological agents and other organisms or the environment” and biosecurity can be defined as “the protection, control, and accountability for valuable biological materials [ … ] within laboratories, to prevent their unauthorized access, loss, theft, misuse, diversion, or intentional release” (WHO, 2006). Biosafety and biosecurity identified the risks that can be posed in active or passive situations by intentional or accidental mistakes of certain experiment practitioners in labs or garages (for garage biology or DIYbio cases). Both the laboratory safety and the garage biology safety constitute the major parts of biosafety regarding SynBio, especially the former one as laboratories are still the major sources of knowledge and material production at present (Douglas and Savulescu, 2010; Thompson, 2012).

*Cluster 4 (green):* This cluster is related to the technologies and upstream science disciplines of SynBio. The high-frequency keywords here are “methods,” “gene drive,” “gene editing,” “Clustered Regularly Interspaced Short Palindromic Repeats,” “systems biology,” “biological engineering,” “metabolic engineering,” and “agricultural biotechnology,” and the issues about technology such as “patents,” “policy,” “bioeconomy,” “biocrime,” and “bioterrorism.” Systems biology, bioengineering, metabolic engineering, biochemistry, and other disciplines and technologies constitute the cornerstone of SynBio. In recent years, accurate, economical, and accessible powerful gene-editing technologies like CRISPR and its upgraded versions have provided significant impetus for the leap-forward development of SynBio. In addition, the research about technology-related intellectual property and patents (Calvert, 2012), as well as policy and regulation issues (Wiek et al., 2012), has also evolved with SynBio.

### 3.4 RQ4: potential research trends of the SynBio social research

A time zone view of the distribution of the literature topics can be used to illustrate the research focus of each period and show the evolution of research topics and then supply some clues to analyze the potential research trends. In this section, we redistributed and re-visualized the keywords of the collected literature published in the last 5 years, as shown in Figure 6, and switch our focal spots to the latest and cutting-edge topics of the current field. Keywords like “public engagement,” “RRI,” “ethics,” “risk,” “biotechnology,” and “governance” still have a high frequency in the last 5 years, and these issues will not fade out soon as they deeply involve human beings. However, instead of the four cluster themes analyzed in RQ3 which would dominate the field in the long term and serve as the mainstream themes for the following years; here, we focus on the three emerging themes that might share some novel perspectives for further research. Although these literature that indicate research trends might not be highly cited yet, the extended framework and concerning topics put forward by them can also be valuable as compared with the long-standing themes mentioned previously*.*

(1) New possibilities and challenges brought by SynBio for the transformation of bioeconomy

The influence of SynBio no longer stays in the fields of basic scientific research or technological development but has penetrated the social and economic fields and demonstrated its considerable economic value and commercial potential. SynBio has been offering possibilities and opportunities for global governments to formulate visions of a transition toward a bioeconomy and has played the enabling and transformational role of entrepreneurship (Kuckertz et al., 2020) and enlightened research and development with its exemplary convergent nature like carefully designed, stakeholder-inclusive, and community-directed evolution in bioeconomy (Bueso and Tangney, 2017). Moreover, the public engagement access offered by SynBio helps recruit and train the future workforce for bioeconomy, such as the competition iGEM and citizen science practices (Warmbrod et al., 2020). However, researchers have also noticed the necessary considerations and points of focus in this endeavor owing to the risks and uncertainties of utility in upcoming deployments outside the labs in bioeconomy practices (Parker and Kunjapur, 2020). Particularly, the self-driven commercialization process would propel the influence of SynBio broadly and rapidly, highlighting the lag of policy and entangling more opposing forces against governance.

(2) The remodeling of SynBio’s multi-dimensional impacts by art

Not many articles discussed the relationship between art and SynBio, but several feasible perspectives have been explored according to the current publications, such as the fusion of art and SynBio in material practices, and the reflection on SynBio with artworks. Merritt et al. (2020) used the novel living media interfaces (LMIs) to show the case of the interaction between biological materials and digital systems as responsive living media and SynBio, as engineered biology, could offer plenty of options for the feasible fusion practice. Vaage (2020) appealed to expand the utility of artworks to provide counter-images to challenge mechanistic assumptions, i.e., the living machines metaphor and the perception of life as controllable in the context of SynBio. Meanwhile, Calvert and Schyfter (2017) argued that “engaging more closely with art and design can enrich STS work by enabling an emergent form of critique” and “open up the science by exploring implicit assumptions and interrogating dominant research agendas.” Above all, art offers an interdisciplinary perspective to examine the impacts of SynBio practically and theoretically, and more interaction approaches and science communication patterns regarding SynBio need to be explored.

(3) The role of knowledge of SynBio

The role of knowledge of SynBio has changed since its inception. Its initial form appeared as the knowledge of a certain scientific discipline, which was mainly disseminated within the scientific community and then gradually diffused to the public and exerted social influences. In this process, not only the products but also the knowledge itself became the object of ethical research. Therefore, its role has evolved from “knowledge for use” to “knowledge for public communication” and “knowledge as an ethical object.” Nordmann (2015) appealed to examine SynBio on “epistemic values, the ethos and authority of science, and the relation of knowledge and power.” Douglas and Savulescu (2010) put forward the concept of “ethics of knowledge” about SynBio based on the “misuse of knowledge” and appealed not to regard the creation and dissemination of knowledge of SynBio as granted or beyond doubt. Instead, it called for attention to the risk of misuse of knowledge and advocated for reflection on it as the object of ethical governance, during which its role had been radically changed.

## 4 Discussion

Based on Professor Shapira et al.’s (2017) research, this paper reconstructed the conceptual system and search strategies of SynBio to retrieve the literature related to it in the sphere of social sciences and humanities and formulated a set of effective retrieval strategies in the social context based on the search result of each related terms. After visualizing the publishing trends in this field, we found that the publications in this field had been growing rapidly since 2006, which encouraged the researchers greatly. In addition, we investigated the top productive countries and regions in this field, which mainly consisted of the developed world. The United States and the United Kingdom had the top two highest numbers of publications, which contained several prolific research teams from universities in regions such as Edinburgh and Manchester. However, this kind of cooperation was basically within the institutions and the inter-institution cooperation was less significant here.

The research areas of the collected literature involved philosophy and ethics mostly, such as the history and philosophy of science, ethics, philosophy, medical ethics, *etc.* In addition, communication, environmental studies, and other social sciences also took a certain proportion, which implied the diversity of research themes about SynBio in a social context. After that, we investigated the intellectual base of the current field and the high-frequency co-cited references and found that knowledge sources of natural sciences and social sciences took the dominant places in the earlier and later stages separately. With cluster analysis and visualization technology, a co-cited reference network was generated and visualized to find the three representative stages of knowledge background and the research topics of each stage, namely, knowledge transfer, public engagement, and diversified reflection. The shift of the stages manifests how scientists, social scientists, and public interact with each other with knowledge creation, transaction, diffusion, and reinvention occurring within respective communities. In public practice, SynBio experiences the diffusion of innovations when spreading among citizens in breadth and the individual engagement from the perception and attitude to behavioral practices in depth. Owing to the engagement of public in SynBio via DIY biology practice, the diffusion of SynBio knowledge *per se* leads to the ethical issues of potential knowledge misuse and simultaneously push forward the epistemological thinking of philosophical researchers rather than the previous insights into the nature, feature, benefits, and risks of SynBio as a discipline and knowledge system. This is where the intellectual base “public engagement” and the potential research trend “the role of knowledge of SynBio” converge and shows the paradigm shift from value and essence to knowledge in the philosophical perspective. In addition, this trend enlightens us and offers theoretical frameworks to review other disciplines and dual-use technologies which invite close public engagement and participation like artificial intelligence, cybersecurity, and autonomous weapons.

Source titles of the collected literature were also listed to help researchers follow the progress and development of this field. We tried to identify hotspots in this area with co-word methodology, managed to cluster the keywords into four domains, and introduced the theme of each domain. These hotspots could help locate the research fronts in the current field, to access novel ideas, and systematically understand the development of the field. Finally, we extracted and visualized the network of keywords in the last 5 years, tried to find out the potential research trend, and identified the three novel research domains, which might inspire the researchers and stakeholders. Apart from the epistemological trend regarding SynBio expatiated previously, the practical trends of SynBio like bioeconomy and art, on the one hand, echo to the third stage of intellectual base “diversified reflections;” on the other hand, it indicates that researchers stride into further exploration of emergent application and corresponding social impacts of SynBio commercialization and of potentials to boost social good.

These relatively positive and optimistic trends of waning ethical vigilance is due to the fact that despite the rapid development of SynBio, no relevant biosafety or biosecurity incident has occurred so far, owing to the persistent normative efforts of research management and policies on emerging technologies. Moreover, it is because of the promising latent and applicational prospect of SynBio in recent years, such as the synthesis of starch (Cai et al., 2021), glucose, and fatty acids (Zheng et al., 2022) from carbon dioxide. Therefore, it might be safe to boldly set about investigating the issues of diversified commercial possibilities, the impact of novel forms of public involvement, and other feasible perspectives within and beyond SynBio field.

## 5 Conclusion

In this study, we constructed a novel conceptual framework and search strategy about SynBio in the context of social sciences and systematically reviewed the relevant literature in this field with mature and well-proven methods such as co-occurrence analysis and knowledge mapping. We also illustrated and clarified the literature from three aspects: hotspots, potential research trends, and intellectual base.

During the COVID-19 pandemic, considerable conspiracies and rumors regarding bioweapon and biohazard leakage have hindered the control of pandemic spread (Romer and Jamieson, 2020; Zhang et al., 2021) and shattered people’s confidence over biotechnologies and institutions. This negative attitude toward biotechnologies aggravated as conspiracies about COVID-19 (accounted as bioweapon) entered collective sense-making (Nadesan, 2022), and a substantial portion of people even endorse contradictory conspiracy theories (Petrović and Žeželj, 2022) However, in these unexpected emerging situations, SynBio can benefit the treatment of infectious diseases and the development of vaccines. SynBio and its methodologies, such as RNA delivery using lipid nanoparticles, have already functioned in the production of COVID-19 RNA vaccines and medicines (Liu et al., 2020) and become one of the fundamental methods for the rapid development of fully synthetic RNA vaccines (Rappuoli et al., 2021) in the post-COVID-19 era. Therefore, in such a complex situation, how to release the positive potentials of SynBio, avoid the risks and dangers it might bring to the world by institutionalized means, and explore appropriate ethical rules and policies to accelerate the benign development of SynBio and benefit human beings has become an inescapable issue. COVID-19 only magnifies the urgency. Thus, in addition to the natural science research, non-natural sciences like social sciences, philosophy, and ethics also need to pay more attention to conduct research in advance before the possible events and the cooperation of natural science scientists, government officers, and stakeholders in society are required (Komen et al., 2020). Moreover, the concrete and practical RRI framework, public engagement through democratic deliberation subject to the post-COVID-19 working manner, and ethics of knowledge in production and diffusion should be taken into the research agenda.

However, this study also has its limitations. For example, regarding data sources, only the records from the SSCI and AHCI were included in this study, even to ensure the impact and authority of the literature. Literature like monographs, book chapters, dissertations, academic reports, non-English literature, and public opinion on media and government, white papers are excluded from the current research in consideration of the consistency of data format and data source for bibliometric practices, but the importance of these literature should not be neglected and deserve to be studied exclusively. Owing to the delays in publishing and the agenda gap between social or ethical research and natural science research, academia and industry, public and researchers, the cutting-edge scientific discoveries, industrial success, and public hotspots do take time to get into social researchers’ vision and gain attention. While the social and ethical research articles cannot cover all even the latest progress, particularly the breakthroughs of leading research teams in China and the US, looking into the long-standing social and ethical issues and the history of the philosophical thinking in research articles regarding SynBio can be an effective trajectory to rethink SynBio. For further research, on the one hand, the sub-areas such as ethics or philosophy can be worthy of further study to explore enlightening ideas. On the other hand, the potential trends proposed in this paper have pointed out possible directions for further research.

## Data Availability

The raw data supporting the conclusion of this article will be made available by the authors, without undue reservation.
